# First Insights into the Genome Sequence of the Strictly Anaerobic Homoacetogenic *Sporomusa sphaeroides* Strain E (DSM 2875)

**DOI:** 10.1128/genomeA.00037-17

**Published:** 2017-03-23

**Authors:** Genis Andrés Castillo Villamizar, Rolf Daniel, Anja Poehlein

**Affiliations:** Genomic and Applied Microbiology and Göttingen Genomics Laboratory, Georg-August University Göttingen, Göttingen, Germany

## Abstract

Here, we report the draft genome sequence of *Sporomusa sphaeroides* strain E (DSM 2875), a strict anaerobic homoacetogenic bacterium. It is able to grow autotrophically on different one-carbon compounds. The strain possesses several genes of the Wood-Ljungdahl pathway. The genome consists of a single chromosome (4.98 Mb).

## GENOME ANNOUNCEMENT

The autotrophic metabolism of diverse acetogenic bacteria is used for the development of economically relevant chemicals such as acetate, ethanol, butyrate, and butanol. Likewise, the quest for alternative, renewable, and sustainable energy sources resulted in an increased interest for processes involving anaerobic digestion. Among the most studied organisms involved in anaerobic digestion processes are Gram-positive acetogens like *Clostridium ljungdahlii*, *C. aceticum*, and the thermophile *Moorella thermoacetica*. Gram-negative acetogens comprise several species of the* Sporomusa* genus (1–3).

The publication of genome sequences of many acetogens involved in biotechnological processes improves knowledge and drives the development of new and more efficient production platforms (4–8). In this study, we report the draft genome sequence of *Sporomusa sphaeroides* E (DSM 2875). This organism has been isolated from mud samples of the German Leine River (9).

A MasterPure complete DNA purification kit (Epicentre, Madison, WI, USA) was used to isolate chromosomal DNA of *S. sphaeroides* E (DSM 2875). The extracted DNA was used to generate 454 shotgun, 454 paired-end, and Illumina shotgun libraries (paired-end) according to the manufacturers’ protocols (Roche Life Sciences, Mannheim, Germany, and Illumina, Inc., San Diego, CA, USA). The libraries were sequenced using a 454 GS-FLX system (Titanium GS70 chemistry; Roche Life Sciences, Mannheim, Germany) and a Genome Analyzer II (Illumina, Inc.). Sequencing resulted in 251,686 454 shotgun reads, 100,698 454 paired-end reads (1.6-kb and 2.8-kb insert sizes), and 7,621,534 Illumina paired-end reads (112 bp). Assembly of the reads using Roche Newbler assembly software 2.6 for scaffolding and MIRA software (10) resulted in 35 scaffolds with 108 contigs. The average coverage was 26.8-fold for 454 and 171.44-fold for Illumina. Some gaps were closed using PCR and Sanger sequencing of the products. Analysis of the obtained sequences was completed using Gap4 (version 4.11) software of the Staden package (11, 12). The final draft genome of *S. sphaeroides* (16 contigs) consists of a single chromosome of 4.98 Mb with an overall G+C content of 47.21%. Gene prediction and annotation were performed using Prokka (13). The draft genome harbored 17 rRNA genes, 88 tRNA genes, 3,564 protein-coding genes with predicted functions, and 1,150 genes coding for hypothetical proteins.

The cluster of genes encoding enzymes of the methyl and carbonyl branches of the Wood-Ljungdahl pathway is present in the genome and showed the same organization as described for *S. ovata* DSM 2662 (6). The genome contains genes encoding for enzymes involved in the metabolism of one-carbon compounds, including *cooS* for the putative synthesis of the carbon monoxide dehydrogenase (CODH) required for growing on CO. Five genes of the formate dehydrogenase (*fdhs*) complex involved in the oxidation of formate were also detected. Finally, the methyltransferase genes *mtaB* and *mtaD*, required for methanol-specific methyl transfer (14), were also present.

### Accession number(s).

This whole-genome shotgun project has been deposited at DDBJ/ENA/GenBank under the accession number LSLJ00000000. The version described in this paper is the first version, LSLJ01000000.
